# Omentin-1 prevents inflammation-induced osteoporosis by downregulating the pro-inflammatory cytokines

**DOI:** 10.1038/s41413-018-0012-0

**Published:** 2018-03-30

**Authors:** Shan-Shan Rao, Yin Hu, Ping-Li Xie, Jia Cao, Zhen-Xing Wang, Jiang-Hua Liu, Hao Yin, Jie Huang, Yi-Juan Tan, Juan Luo, Ming-Jie Luo, Si-Yuan Tang, Tuan-Hui Chen, Ling-Qing Yuan, Er-Yuan Liao, Ran Xu, Zheng-Zhao Liu, Chun-Yuan Chen, Hui Xie

**Affiliations:** 1 Movement System Injury and Repair Research Center, Xiangya Hospital, Central South University, Changsha, Hunan 410008 China; 2 Department of Spine Surgery, Xiangya Hospital, Central South University, Changsha, Hunan 410008 China; 3 Department of Orthopedics, Xiangya Hospital, Central South University, Changsha, Hunan 410008 China; 4 Department of Forensic Science, School of Basic Medical Sciences, Central South University, Changsha, Hunan 410013 China; 5 Xiangya Nursing School, Central South University, Changsha, Hunan 410013 China; 6 Second Xiangya Hospital, Central South University, Changsha, Hunan 410011 China; 7 Hunan Key Laboratory of Organ Injury, Aging and Regenerative Medicine, Changsha, Hunan 410008 China; 8 Department of Sports Medicine, Xiangya Hospital, Central South University, Changsha, Hunan 410008 China; 9 National Clinical Research Center for Geriatric Disorders, Xiangya Hospital, Central South University, Changsha, Hunan 410008 China; 10 China Orthopedic Regenerative Medicine Group (CORMed), Changsha, Hunan China

## Abstract

Osteoporosis is a frequent complication of chronic inflammatory diseases and increases in the pro-inflammatory cytokines make an important contribution to bone loss by promoting bone resorption and impairing bone formation. Omentin-1 is a newly identified adipocytokine that has anti-inflammatory effects, but little is known about the role of omentin-1 in inflammatory osteoporosis. Here we generated global *omentin-1* knockout (*omentin-1*^−/−^) mice and demonstrated that depletion of omentin-1 induces inflammatory bone loss-like phenotypes in mice, as defined by abnormally elevated pro-inflammatory cytokines, increased osteoclast formation and bone tissue destruction, as well as impaired osteogenic activities. Using an inflammatory cell model induced by tumor necrosis factor-α (TNF-α), we determined that recombinant omentin-1 reduces the production of pro-inflammatory factors in the TNF-α-activated macrophages, and suppresses their anti-osteoblastic and pro-osteoclastic abilities. In the magnesium silicate-induced inflammatory osteoporosis mouse model, the systemic administration of adenoviral-delivered omentin-1 significantly protects from osteoporotic bone loss and inflammation. Our study suggests that omentin-1 can be used as a promising therapeutic agent for the prevention or treatment of inflammatory bone diseases by downregulating the pro-inflammatory cytokines.

## Osteoporosis: Fat-secreted protein guards against bone loss in mice

An anti-inflammatory molecule produced by fat tissue helps protect against bone loss in mouse model of osteoporosis. Researchers from China’s Central South University in Changsha, led by Hui Xie, examined whether omentin-1, a protein secreted by fat tissue recently implicated in the development of obesity and diabetes, might play a role in osteoporosis as well. In mice engineered to lack omentin-1, they observed more bone tissue destruction and increased numbers of bone-resorbing osteoclast cells than in normal, healthy animals. The researchers also administered exogenous omentin-1 to mice either with an overactive inflammatory molecule or experimentally induced to develop osteoporosis. In both cases, they found that the protein suppressed inflammation and guarded against bone loss. Omentin-1 thus provides promising therapeutic agent for preventing or treating inflammatory bone diseases such as osteoporosis.

## Introduction

Bone remodeling is a lifelong process in vertebrates that relies on the balance between bone formation by osteoblasts and bone resorption by osteoclasts.^1–3^ When the balance is disrupted, as is seen in chronic inflammatory diseases, such as rheumatoid arthritis and ankylosing spondylitis, abnormal bone loss and osteoporotic fractures often occurs.^4^ Studies have reported that increases in the pro-inflammatory cytokines during chronic inflammation make an important contribution to bone loss by promoting osteoclast differentiation and/or inhibiting osteoblast maturation and function.^1,4^ Thus, strategies designed to inhibit pro-inflammatory activities hold promise in promoting bone regeneration and preventing inflammation-induced osteoporosis.

Omentin-1, also referred to as intelectin-1 (ITLN1), is a novel adipocytokine that is highly expressed in human visceral fat tissue and in mouse small intestine. This protein is also highly abundant in plasma and circulating omentin-1 concentration has been found to be inversely correlated with the levels of pro-inflammatory factors, such as interleukin (IL)-6 and tumor necrosis factor-alpha (TNF-α) in individuals with impaired glucose regulation and chronic artery disease.^5,6^ Recently, it has been reported that omentin-1 can inhibit pulmonary inflammation and endothelial injury after lipopolysaccharide (LPS)-induced acute respiratory distress syndrome (ARDS) in mice.^7^ Moreover, omentin-1 can attenuate atherosclerotic lesion formation in apolipoprotein E-deficient mice by reducing the inflammatory response of macrophages.^8^ However, up to now, few studies have investigated the role of omentin-1 in inflammation-induced osteoporosis.

Previously, we demonstrated that omentin-1 could inhibit osteoclast differentiation in vitro via an indirect mechanism by stimulating the production of osteoprotegerin (OPG) and reducing the secretion of receptor activator for nuclear factor κB ligand (RANKL) in osteoblasts.^9^ We also determined in vivo that intravenous administration of adenoviral-delivered omentin-1 could prevent bone loss and enhance bone strength in ovariectomy-induced osteoporotic mice,^9^ as well as in OPG-deficient mice.^10^ Based upon these evidences outlined above, we hypothesized that omentin-1 might have inhibitory effects on inflammation-induced osteoporosis, as it may target both the bone metabolism and the inflammatory process.

In this study, we firstly generated global *omentin-1* knockout (*omentin-1*^−/−^) mice for determining the role of omentin-1 in inflammation and bone loss. We also assessed the effects of recombinant omentin-1 on the TNF-α-induced inflammatory responses of macrophages, as well as on the activated macrophages-secreted factors-mediated regulation of osteoblast differentiation and osteoclast formation in vitro. Moreover, we explored the therapeutic potential of adenovirus-mediated delivery of omentin-1 in a mouse model of inflammation-induced osteoporosis. Our study aimed to uncover the role of omentin-1 in inflammatory osteoporosis and to preliminarily elucidate the mechanism underlying this process.

## Results

### Omentin-1 depletion induces inflammatory bone loss-like phenotypes in vivo

**Fig. 1:**
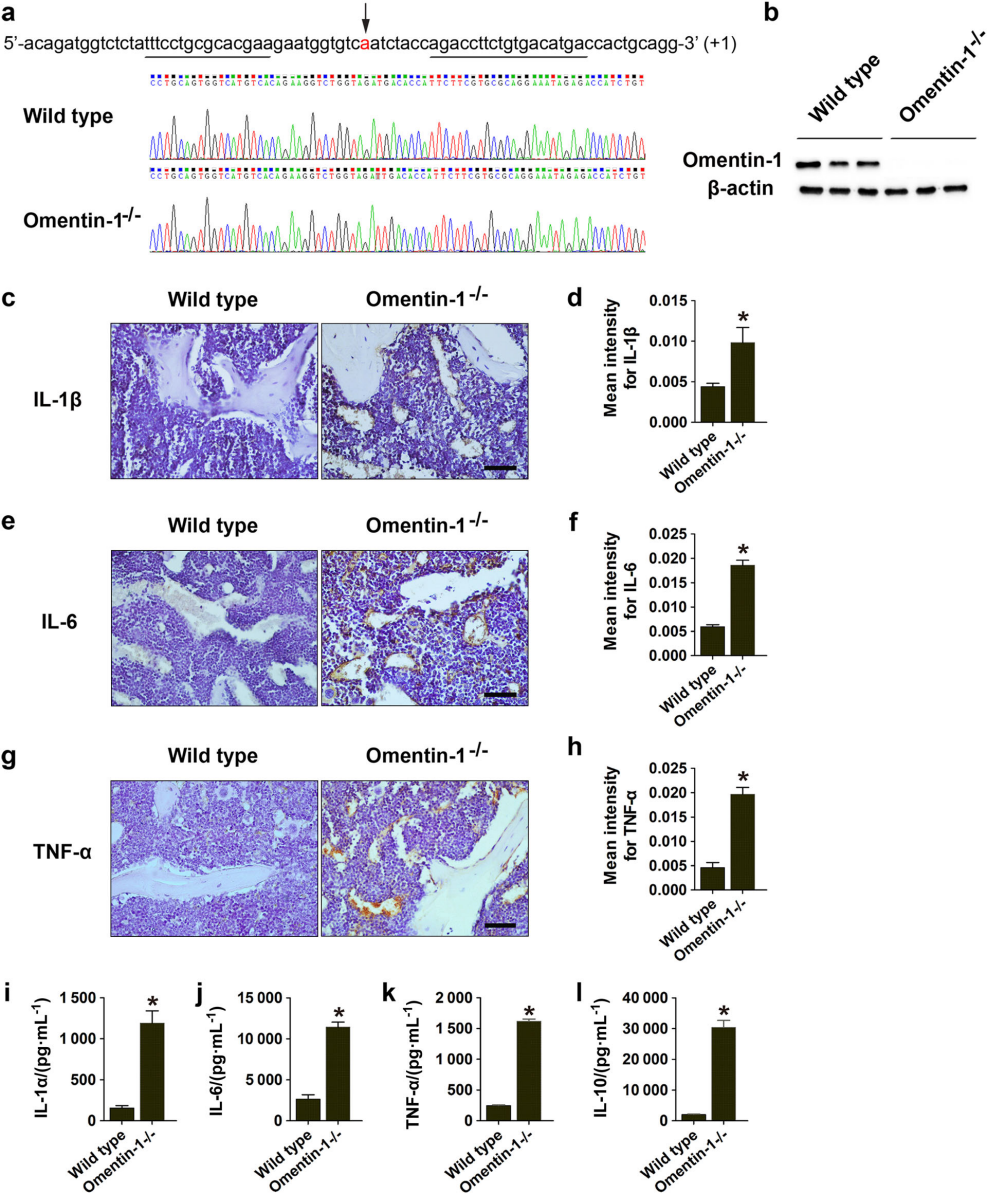
Omentin-1 depletion induces inflammatory responses in mice. **a**, **b** One base (**a**) was inserted into the sequence of *omentin-1* gene on exon 4 in one strand, resulting in the early termination of omentin-1 protein translation. Genomic DNA from tail tips of *omentin-1*^−/−^ mice and their wild-type littermates was genotyped by DNA sequencing (**a**). The depletion of omentin-1 in *omentin-1*^−/−^ mice was verified by western blot analysis of omentin-1 protein in the small intestines from *omentin-1*^−/−^ mice and their wild-type littermates. *n* = 3 per group (**b**). **c**–**h** Representative images of immunohistochemical staining for IL-1β (*n* = 6 or 9 per group), IL-6 (*n* = 4 or 6 per group) and TNF-α (*n* = 6 or 9 per group) in femora of *omentin-1*^−/−^ and wild-type mice. The graphs show mean intensity for the positively stained areas. Scale bar: 50 μm. **i**–**l** The serum concentrations of pro-inflammatory cytokines IL-1α, IL-6, and TNF-α (**i**–**k**) and the anti-inflammatory factor IL-10 (**l**) in *omentin-1*^−/−^ and wild-type mice, as determined by ELISA. *n* = 6 or 9 per group. Data are shown as the mean ± s.d. *P < 0.05 compared with the control (wild-type) mice.

To confirm the role of omentin-1 in inflammation and bone loss, we generated *omentin-1*^−/−^ mice and characterized the knockout mice by DNA sequencing analysis (Fig. 1a) and western blotting (Fig. 1b). The results revealed that a base (A) was successfully inserted into the sequence of omentin-1 gene and caused a depletion of omentin-1 protein in small intestines (the organ showing the highest expression of omentin-1 in mice^11,12^) of *omentin-1*^−/−^ mice compared with their wild-type littermates. We then evaluated whether omentin-1 deficiency resulted in dysregulated inflammation. Immunostaining revealed a drastic increase in the areas positively stained for the pro-inflammatory mediators, including IL-1β, IL-6, and TNF-α in the bone tissue of *omentin-1*^−/−^ mice when compared with wild-type mice (Fig. 1c–h). Consistently, omentin-1 depletion also caused a remarkable increase in serum concentrations of the IL-1α, IL-6, and TNF-α, as determined by enzyme-linked immunosorbent assay (ELISA) (Fig. 1i–k). Paradoxically, we found that the level of IL-10, an anti-inflammatory cytokine that can suppress osteoclastogenesis and bone resorption,^13,14^ was also markedly elevated in *omentin-1*^−/−^ mice compared to the control mice (Fig. 1l), indicating that omentin-1 depletion leads to dysregulated inflammatory responses in mice.

**Fig. 2:**
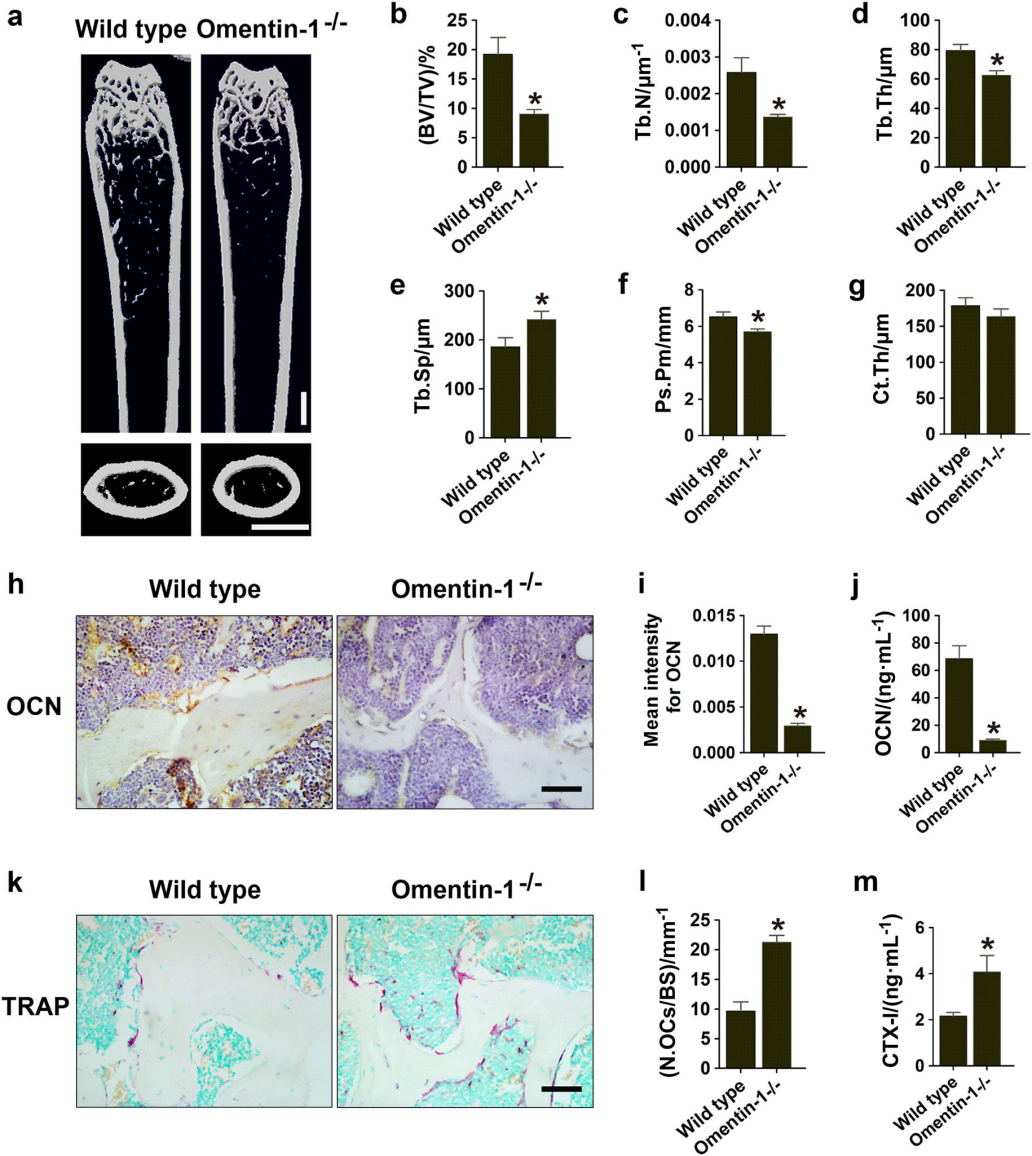
Omentin-1 depletion leads to abnormal bone loss in mice. **a** Representative microcomputed tomography (µCT) images of femora in *omentin-1*^−/−^ and wild-type mice. Scale bar: 1 mm. **b**–**g** Quantitative analyses of the trabecular bone fraction (Tb.BV/TV), trabecular thickness (Tb.Th), trabecular number (Tb.N), trabecular separation (Tb.Sp), periosteal perimeter (Ps.Pm) and cortical bone thickness (Ct.Th) of femora. *n* = 6 per group. **h** Representative images of immunohistochemical staining for OCN. Scale bar: 50 μm. **i** Quantitative analysis of the mean intensity for the positively stained areas in **h**. *n* = 5 or 6 per group. **j** ELISA of the serum concentration of OCN. *n* = 5 or 7 per group. **k** TRAP staining of femora in *omentin-1*^−/−^ and wild-type mice. Scale bar: 50 μm. **l** Quantitative analysis of the number of TRAP^+^ cells in **k**. *n* = 5 or 6 per group. BS bone surface, OCs osteoclasts. **m** ELISA of the serum concentration of CTX-I. *n* = 10 per group. Data are shown as the mean ± s.d. *P < 0.05 compared with the wild-type mice.

Subsequently, microcomputed tomography (µCT) scanning was carried out to evaluate the impact of omentin-1 deficiency on bone mass and microarchitecture in mice. As shown in Fig. 2a, omentin-1 depletion caused a significant trabecular bone loss compared to wild-type mice. Further quantitative analysis determined that *omentin-1*^−/−^ mice displayed remarkably reduced trabecular bone volume fraction, thickness, and number, and increased trabecular separation than that in wild-type mice (Fig. 2b–e). The periosteal perimeter of femoral diaphysis in *omentin-1*^−/−^ mice were also much lower than that of wild-type mice (Fig. 2a, f). Cortical bone thickness was also reduced in *omentin-1*^−/−^ mice compared to wild-type mice, but the difference was not statistically significant (Fig. 2a, g). Immunohistochemical staining and ELISA assay for osteocalcin (OCN), a marker expressed during osteogenic differentiation and mineralization,^15,16^ revealed that osteogenic responses in *omentin-1*^−/−^ mice were markedly decreased compared to wild-type mice (Fig. 2h–j). However, osteoclast activities were dramatically enhanced after omentin-1 depletion, as evidenced by tartrate-resistant acid phosphatase (TRAP) staining on bone tissue sections and ELISA test for the bone resorption marker C-terminal telopeptides of type I collagen (CTX-I) in serum (Fig. 2k–m). We also asked whether omentin-1 deficiency affected the proliferation of osteoblast precursors and osteoclast progenitors by colony-forming unit-fibroblast (CFU-F) and colony- forming units-granulocyte/macrophage (CFU-GM) assays. The results revealed that bone marrow cells from *omentin-1*^−/−^ mice formed much lower numbers of CFU-Fs and higher numbers of CFU-GMs than that in wild-type mice (Supplementary Figure 1), indicating that depletion of omentin-1 impairs the proliferation of osteoblast precursors and enhances the amplification of osteoclast progenitors. Our data suggest that omentin-1 is required for maintaining normal bone metabolism.

We also evaluated body weight, daily consumption of food and water, serum levels of function indicators of liver (ALT and AST) and kidney (creatinine and blood urea nitrogen), blood glucose levels, as well as the serum levels of insulin and iron in *omentin-1*^−/−^ mice and their wild-type littermates (Supplementary Figure 2). The results showed that ALT and AST were increased in *omentin-1*^−/−^ mice compared with wild-type mice, but only by trend. There were also no significant differences in other parameters between *omentin-1*^−/−^ and wild-type mice, indicating that the corresponding function may not be affected by omentin-1 depletion.

### Omentin-1 impairs TNF-α-induced production of pro-inflammatory factors in macrophages

**Fig. 3:**
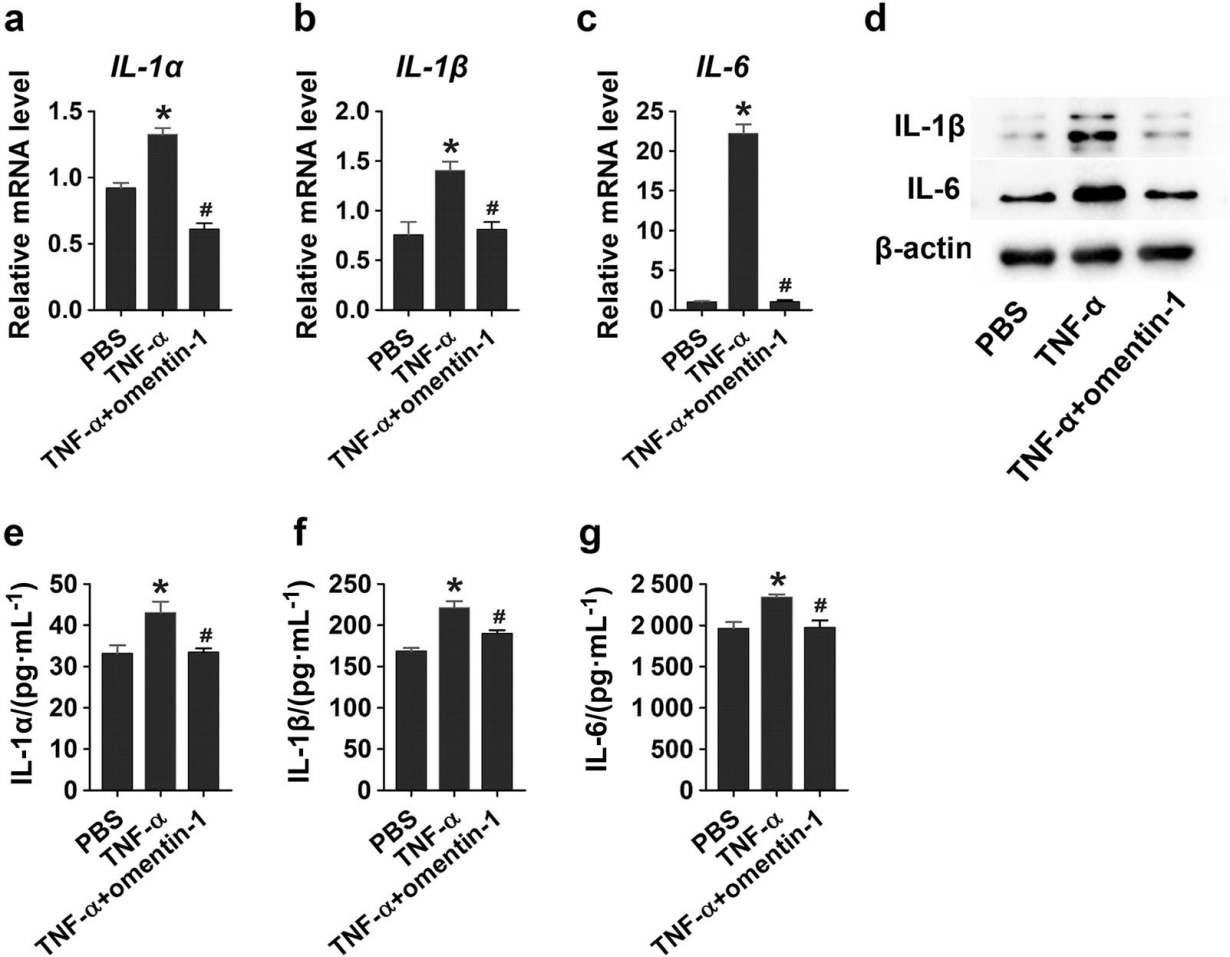
Omentin-1 impairs TNF-α-induced inflammation in macrophages. **a**–**c** RAW264.7 macrophages were treated with 10 ng·mL^-1^ TNF-α or an equal volume diluents (PBS) for 30 min. The culture medium was replaced to remove residual TNF-α and the cells were then incubated in fresh medium supplemented with 300 μg·mL^-1^ omentin-1 or PBS for 24 h. The mRNA levels of *IL-1α*, *IL-1β*, and *IL-6* in RAW264.7 macrophages receiving different treatments were tested by qRT-PCR analysis. *n* = 3 per group. **d** Detection of protein levels of IL-1β and IL-6 in macrophages receiving different treatments. **e**–**g** The concentrations of IL-1α, IL-1β, and IL-6 in conditioned media (CM) derived from macrophages receiving different treatments. *n* = 3 per group. All the results are representative of three independent experiments. Data are shown as the mean ± s.d. *P < 0.05 compared with the PBS group, ^**#**^*P* < 0.05 compared with the TNF-α group.

Given that macrophages are important mediators of inflammation and can release abundant inflammatory factors that contribute to osteoporosis,^17^ we then established an inflammatory macrophage model by treating RAW264.7 cells with TNF-α for 30 min. To confirm the effects of omentin-1 on inflammatory responses of macrophages, the cells were then incubated in fresh culture medium supplemented with omentin-1 or an equal volume of diluents (PBS). Twenty-hours later, quantitative real-time PCR (qRT-PCR) analysis was carried out to detect the mRNA levels of *IL-1α*, *IL-1β*, and *IL-6* in RAW264.7 cells. As shown in Fig. 3a–c, TNF-α significantly enhanced the expression of these pro-inflammatory mediators, but their upregulation was inhibited after the cells were additionally treated with omentin-1. We also performed western blotting to assess the protein levels of IL-1β and IL-6. The result showed that incubation with TNF-α induced a significant increase in the protein production of IL-1β and IL-6, whereas omentin-1 treatment dramatically blocked this effect induced by TNF-α (Fig. 3d). Consistent with these data, ELISA revealed that omentin-1 could potently inhibit TNF-α-induced production of IL-1α, IL-1β, and IL-6 in the culture media of RAW264.7 cells (Fig. 3e–g), indicating that omentin-1 is able to inhibit the activated macrophages to secrete pro-inflammatory mediators.

### Omentin-1 downregulates the anti-osteoblastic and pro-osteoclastic abilities of TNF-α-activated macrophages

**Fig. 4:**
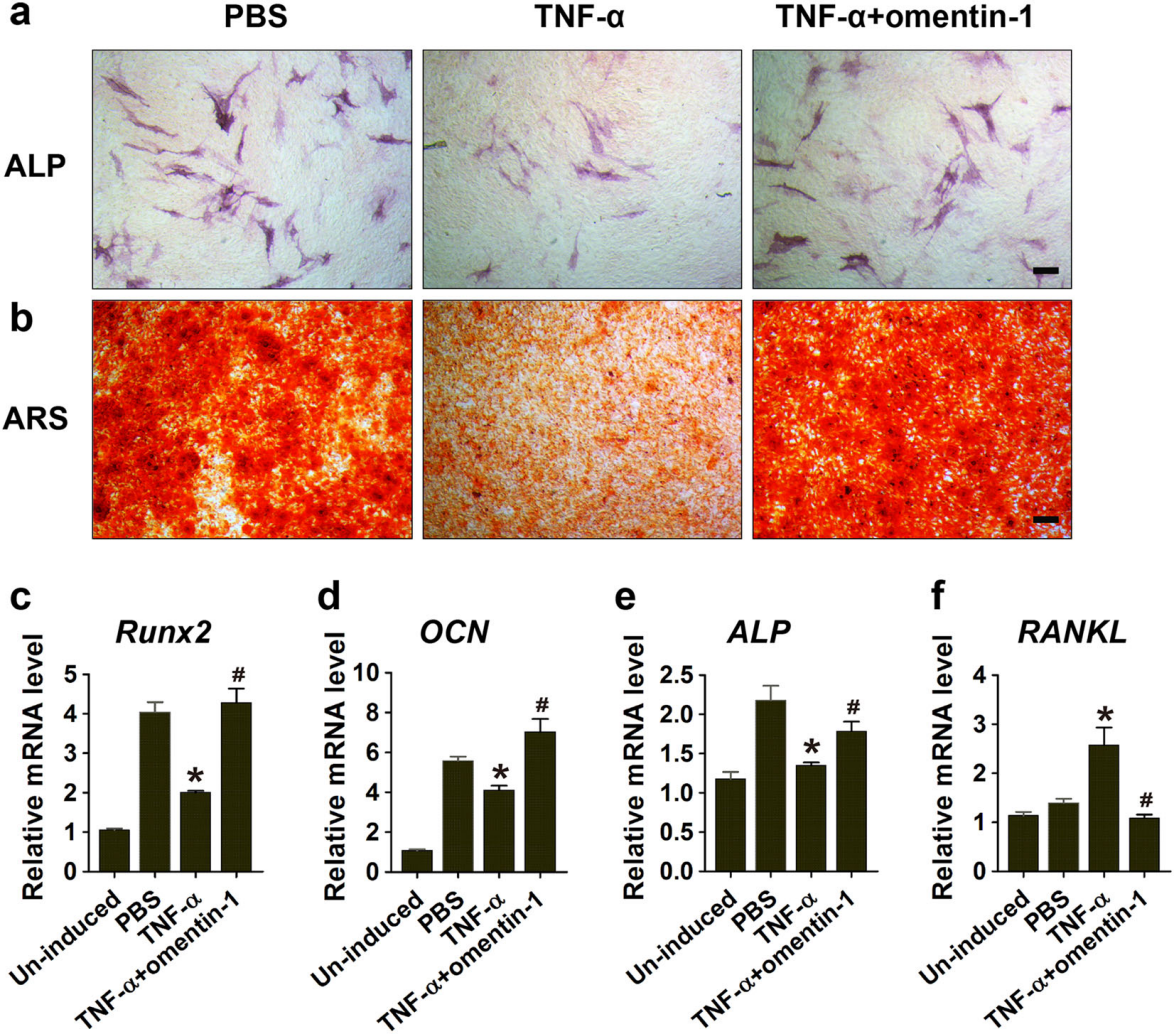
Omentin-1 inhibits the anti-osteoblastic effects of TNF-α-activated macrophages. **a**, **b** MSCs were cultured in osteogenic medium supplemented with the CM from macrophages treated with PBS (30 min + 24 h), TNF-α (10 ng·mL^-1^; 30 min), or TNF-α (10 ng·mL^-1^; 30 min) + omentin-1 (300 μg·mL^-1^; 24 h). Alkaline phosphatase (ALP) (**a**) and alizarin red S (ARS) staining (**b**) were performed after 3 and 14 days of induction, respectively. *n* = 3 per group. Scale bar: 100 μm. **c**–**f** The expression levels of *Runx2*, *OCN*, *ALP*, and *RANKL* were detected by qRT-PCR analyses on day 7. *n* = 3 per group. All the results are representative of three independent experiments. Data are shown as the mean ± s.d. *P < 0.05 compared with the PBS group (cells treated with CM from inactivated macrophages), ^#^*P* < 0.05 compared with the TNF-α group (cells treated with CM from TNF-α-activated macrophages).

To explore whether omentin-1 could inhibit the activated macrophages producing soluble factors to suppress osteogenesis, marrow stromal cells (MSCs) were cultured in osteogenic medium supplemented with the conditioned media (CM) from RAW264.7 macrophages stimulated by TNF-α or TNF-α + omentin-1. Alkaline phosphatase (ALP) and alizarin red S (ARS) staining were performed after 3 and 14 days of differentiation, respectively. The results revealed that the ALP activity (Fig. 4a) and calcium mineral deposition (Fig. 4b) in the differentiated MSCs were attenuated by the TNF-α-activated macrophages-derived CM. However, the effects were not observed in the cells treated with CM from TNF-α + omentin-1-stimulated macrophages. qRT-PCR revealed that the mRNA levels of *Runx2*, *OCN*, and *ALP* were markedly reduced after exposure to TNF-α-activated macrophages-derived CM, whereas the anti-osteoblastic effect of CM was blocked when the activated macrophages were treated with omentin-1 (Fig. 4c–e). We also detected the expression of *RANKL*, a key mediator of osteoclast formation. The result showed a prominent increase in *RANKL* mRNA level in the differentiated MSCs treated with the TNF-α-activated macrophages-derived CM, but the TNF-α + omentin-1-stimulated macrophages-derived CM did not exhibit this effect (Fig. 4f). Our results suggest that omentin-1 cannot only block the negative effect of activated macrophages on osteogenesis, but can also reduce their pro-osteoclastic activity by inhibiting RANKL production in osteoblasts.

**Fig. 5:**
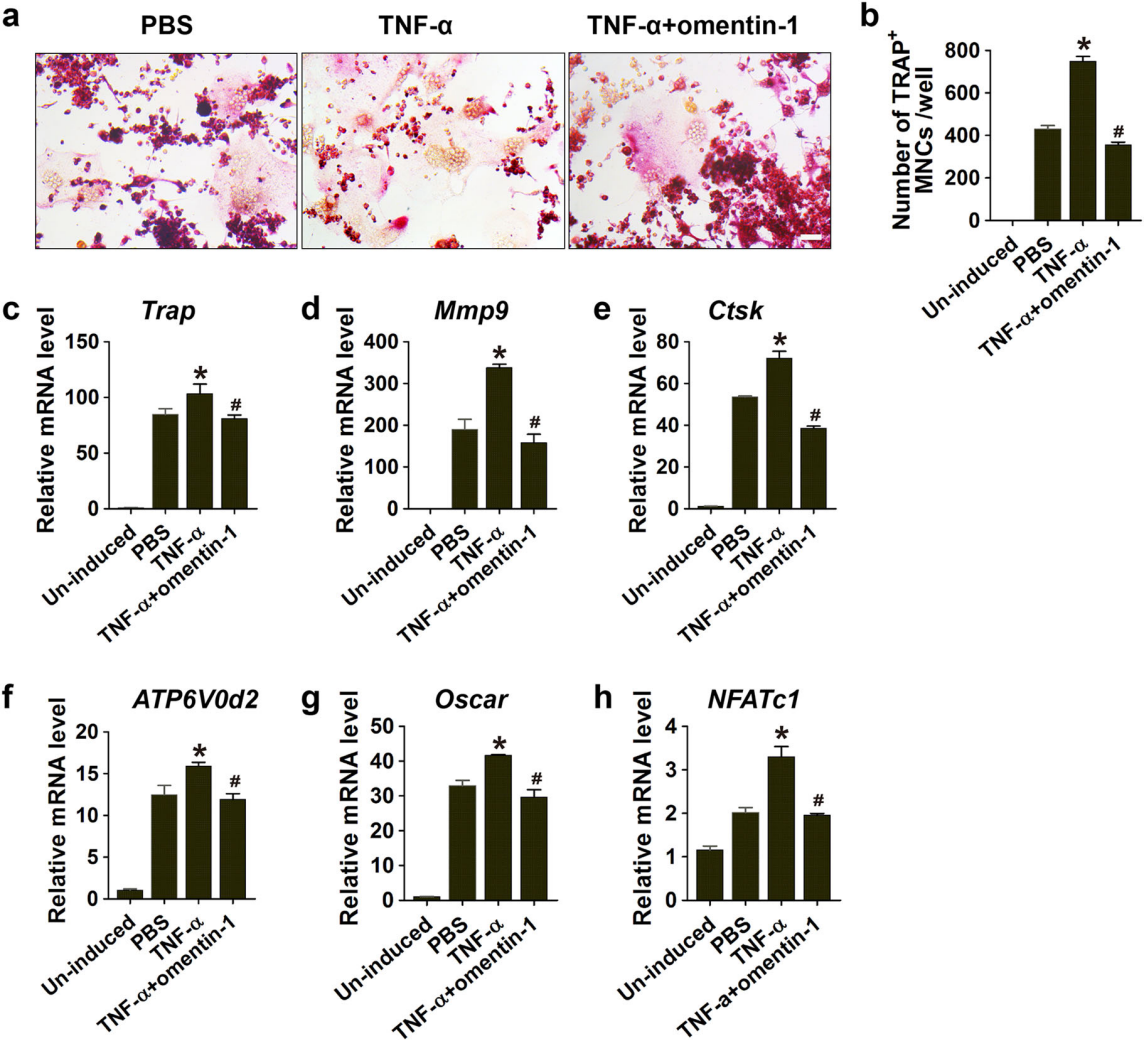
Omentin-1 downregulates the pro-osteoclastic abilities of TNF-α-activated macrophages. **a** TRAP staining of RAW264.7 cells cultured in RANKL-containing medium supplemented with CM from macrophages treated with PBS (30 min + 24 h), TNF-α (10 ng·mL^-1^; 30 min), or TNF-α (10 ng·mL^-1^; 30 min) + omentin-1 (300 μg·mL^-1^; 24 h). Scale bar: 50 μm. **b** Quantitative analysis of the number of TRAP^+^ cells in **a**. *n* = 3 per group. **c**–**h** The expression levels of osteoclastogenesis-related genes including *Trap*, *Mmp9*, *Ctsk*, *ATP6V0d2*, *Oscar*, and *NFATc1* were assessed by qRT-PCR analyses. *n* = 3 per group. All the results are representative of three independent experiments. Data are shown as the mean ± s.d. *P < 0.05 compared with the PBS group (cells treated with CM from inactivated macrophages), ^#^*P* < 0.05 compared with the TNF-α group (cells treated with CM from TNF-α-activated macrophages).

We then asked whether omentin-1 had the potential to directly prevent the activated macrophages to promote osteoclastogenesis. As evidenced by TRAP staining, the CM derived from TNF-α-treated macrophages dramatically augmented the osteoclast differentiation of RAW264.7 cells induced by RANKL; whereas, the effect was inhibited after the activated macrophages were exposed to omentin-1 (Fig. 5a, b). We also performed qRT-PCR analysis to assess the expression of *Trap*, *Mmp9*, *Ctsk*, *ATP6V0d2*, and *Oscar*, which are involved in osteoclast formation and function,^18^ as well as the level of *NFATc1*, a key modulator during the transformation of osteoclast precursors into bone-resorbing osteoclasts.^18^ As shown in Fig. 5c–h, the expression of these osteoclastogenesis-related genes was markedly upregulated in the differentiated RAW264.7 cells treated with the TNF-α-activated macrophages- derived CM compared to the controls. Once the activated macrophages were treated with omentin-1, the pro-osteoclastic effect of their CM was abolished, indicating that omentin-1 can target macrophages to inhibit osteoclast formation.

### Omentin-1 attenuates magnesium silicate-induced inflammation

**Fig. 6:**
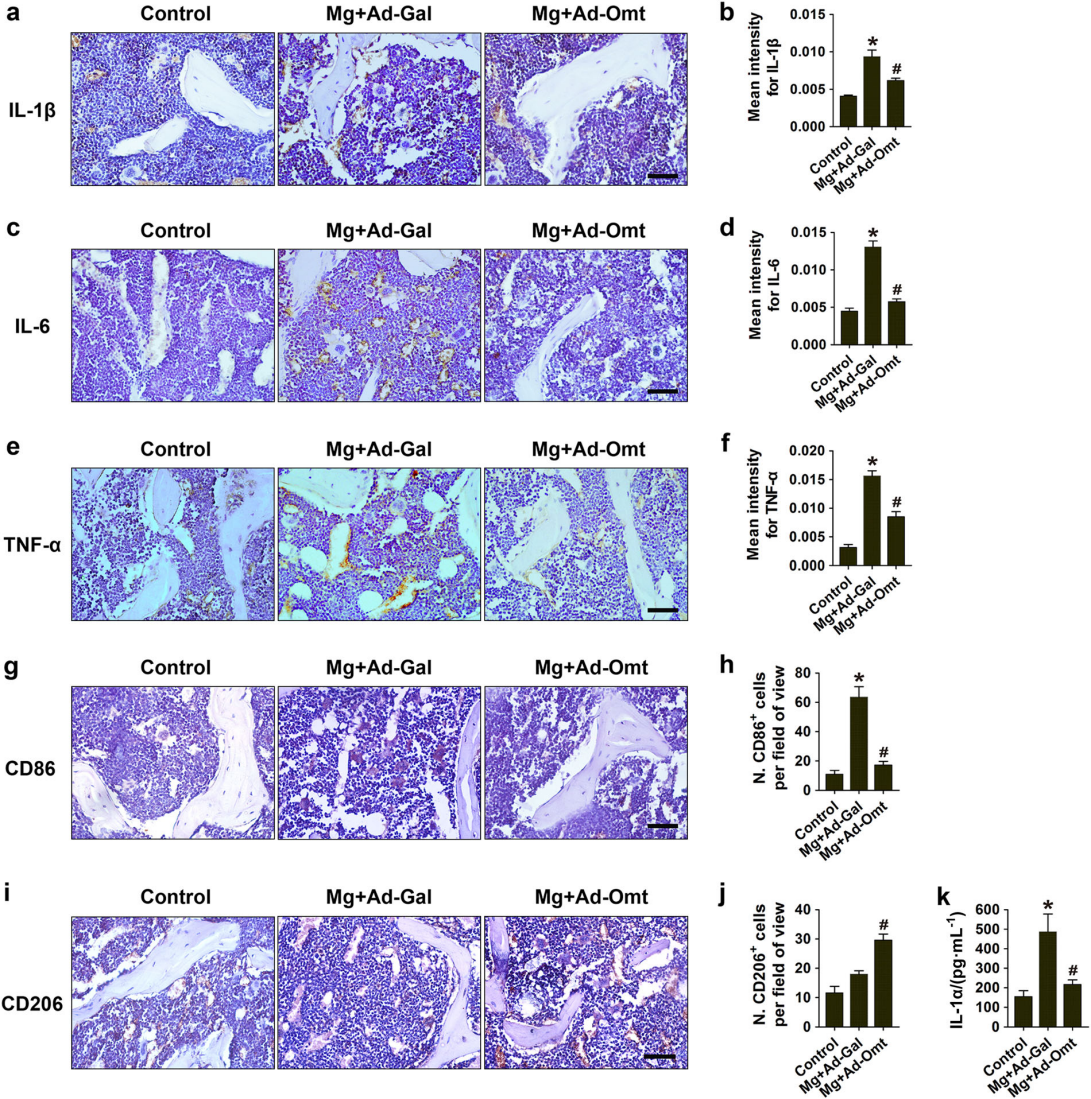
Omentin-1 attenuates inflammatory responses induced by magnesium silicate. **a**–**c** Immunohistochemical staining of IL-1β, IL-6, and TNF-α in bone marrow and on bone surface in mice receiving different treatments. Scale bar: 50 μm. **d**–**f** Quantitative analyses of the mean intensity of the positively stained areas in **a**–**c**. *n* = 5–7 per group. **g** Immunohistochemical staining for CD86 (a marker for pro-inflammatory M1 macrophages). Scale bar: 50 μm. **h** Quantitative analysis of the numbers of CD86^+^ cells in **g**. *n* = 3 per group. **i** Immunohistochemical staining for CD206 (a marker for anti-inflammatory M2 macrophages). Scale bar: 50 μm. **j** Quantitative analysis of the numbers of CD206^+^ cells in **i**. *n* = 3 per group. **k** The serum concentration of IL-1α in different treatment groups, as analyzed by ELISA. *n* = 5–9 per group. Data are shown as the mean ± s.d. *P < 0.05 compared with the control group, ^#^*P* < 0.05 compared with the magnesium silicate + Ad-Gal (Mg + Ad-Gal) group.

Magnesium silicate creates a pro-inflammatory environment with elevated levels of cytokines that contribute to osteopenia and osteoporosis. To test whether omentin-1 could inhibit the magnesium silicate-induced inflammation, experimental models of inflammatory osteoporosis were established by subcutaneous injection of magnesium silicate into the mice, followed by intravenous administration of adenovirus encoding omentin-1 (Ad-Omt) or control adenovirus encoding β-galactosidase (Ad-Gal). Twenty-one days later, the overexpression efficiency of Ad-Omt was confirmed by ELISA assay for serum omentin-1 (Supplementary Figure 3). Immunohistochemistry staining was performed to detect the expression of inflammatory mediators in the bone tissues of mice (Fig. 6a–f). The results showed that magnesium silicate treatment induced a prominent increase in the areas positively stained for IL-1β, IL-6, and TNF-α in bone marrow and on bone surface, as compared to the control group. However, only small positively stained areas were observed in mice co-treated with Ad-Omt. ELISA data revealed that the circulating levels of IL-1α were much higher in magnesium silicate-treated mice than that in control group, but it’s upregulation was attenuated by omentin-1 overexpression (Fig. 6k), indicating a powerful inhibitory role of omentin-1 in inflammation. The levels of serum IL-6 and TNF-α were also enhanced after magnesium silicate treatment and reduced by Ad-Omt, but the differences were not statistically significant (Supplementary Figure 4).

In response to stimuli from the microenvironment, tissue macrophages can be polarized into two distinct functional phenotypes: the classically activated pro-inflammatory M1 phenotype and the alternatively activated anti-inflammatory M2 phenotype associated with tissue remodeling.^19^ We performed immunostaining for CD86 (a marker for M1 macrophages) and CD206 (a marker for M2 macrophages)^20^ to detect the presence of macrophages in the bone tissues of mice. The results showed that CD86-postive M1 macrophages were significantly enhanced after magnesium silicate injection, but Ad-Omt treatment markedly reduced the effect (Fig. 6g, h), suggesting that omentin-1 is able to inhibit the magnesium silicate-induced increase of pro-inflammatory macrophages. CD206 staining revealed that M2 macrophages in magnesium silicate-treated mice were also increased than that in control group but with no significant difference (Fig. 6i, g). Notably, much higher amount of M2 macrophages was observed when magnesium silicate-treated mice were additionally treated with Ad-Omt (Fig. 6i, g), indicating that omentin-1 has the ability to enhance the formation of anti-inflammatory macrophages.

### Omentin-1 prevents inflammatory bone loss induced by magnesium silicate

**Fig. 7:**
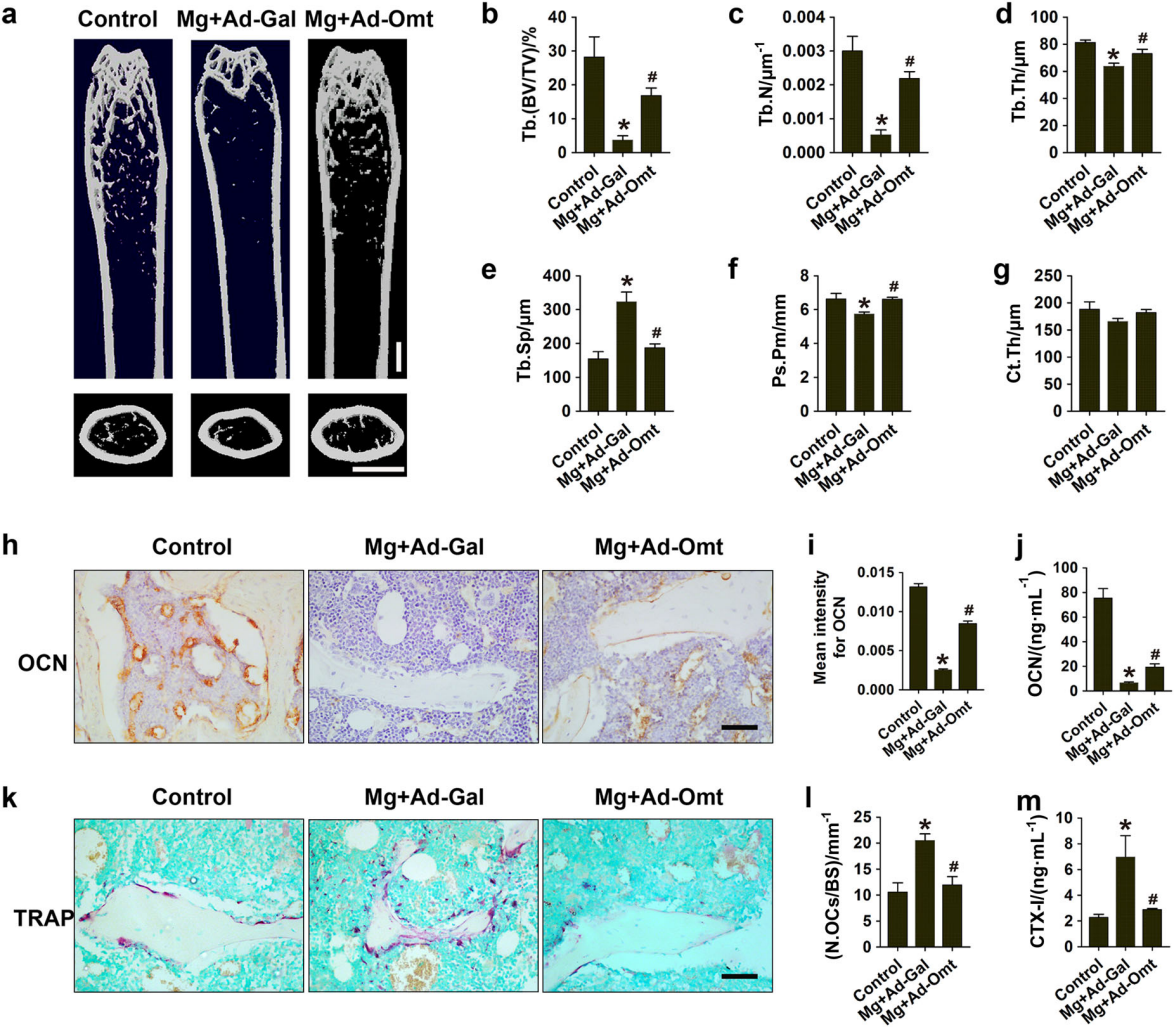
Omentin-1 prevents magnesium silicate-induced inflammatory bone loss. **a** Representative µCT images of femora in mice receiving different treatments. Scale bar: 1 mm. **b**–**g** Quantitative analyses of Tb.BV/TV, Tb.Th, Tb.N, Tb.Sp, Ps.Pm, and Ct.Th of femora in **a**. *n* = 6–8 per group. **h** Representative images of immunohistochemical staining for OCN. Scale bar: 50 μm. **i** Quantitative analysis of the mean intensity of OCN positively stained areas in **h**. *n* = 5–6 per group. **j** ELISA of the serum concentration of OCN. *n* = 6–8 per group. **k** TRAP staining of femora in mice receiving different treatments. Scale bar: 50 μm. **l** Quantitative analysis of the number of TRAP^+^ cells in **k**. *n* = 6–7 per group. **m** ELISA of the serum concentration of CTX-I. *n* = 6–7 per group. The data are shown as the mean ± s.d. *P < 0.05 compared with the control group, ^#^*P* < 0.05 compared with the Mg + Ad-Gal group.

We next examined the effects of omentin-1 on inflammation-induced bone loss. Figure 7a shows µCT images with greatly decreased femoral trabecular bone content in magnesium silicate-treated mice compared with control mice, whereas adenoviral delivery of omentin-1 markedly mitigated the effect. Quantitative measurements revealed that Ad-Omt prevented the magnesium silicate-induced reduction of trabecular bone volume fraction, thickness, and number, as well as improved the trabecular separation in the femur (Fig. 7b–e), which further confirmed the preventive efficacy of omentin-1 on inflammatory bone loss. There was no significant difference in cortical bone thickness between groups, but the decrease of periosteal perimeter induced by magnesium silicate was found to be inhibited by Ad-Omt (Fig. 7a, f, g), suggesting a positive role of omentin-1 in cortical bone formation. Consistently, OCN immunostaining and ELISA assay for serum OCN revealed that the osteogenic response was decreased after magnesium silicate injection, but this effect was inhibited by Ad-Omt (Fig. 7h–j). TRAP staining of femur sections showed a large amount of TRAP^+^ cells on the trabecular bone surface in magnesium silicate-treated mice; whereas, the numbers of TRAP^+^ cells were markedly reduced in mice additionally treated with Ad-Omt (Fig. 7k, l). ELISA test for serum CTX-I indicated that omentin-1 overexpression blocked the magnesium silicate-induced increase of bone resorption activity (Fig. 7m). As evidenced by CFU-F and CFU-GM assays, overexpression of omentin-1 could reverse the magnesium silicate-mediated inhibition of proliferation of osteoblast precursors and stimulation of proliferation of osteoclast progenitors (Supplementary Figure 5). Our results suggest that omentin-1 is able to rescue the impaired osteogenic potential and suppress osteoclast overproduction induced by inflammation.

## Discussion

Osteoporosis is a frequent complication of chronic inflammatory diseases and affects millions of aging populations worldwide.^21–23^ Chronic inflammation associated with aging and arthritis promotes bone resorption and impairs bone formation.^24^ Majority of the drugs currently used in osteoporosis are anti-resorptive agents, but cannot restore the significant bone loss that has already occurred at the time of diagnosis.^24^ Thus, a better therapeutic strategy for osteoporosis would not only address issues with bone homeostasis, but also control local inflammation. Omentin-1 is a newly identified adipocytokine and plays functional roles in inflammation, metabolic disorders and cardiovascular diseases.^6–8,25–29^ Our previous studies revealed that omentin-1 could inhibit osteoclast differentiation and ameliorate estrogen deficiency-induced bone loss by downregulating osteoblast RANKL/OPG ratio.^9^ These features make omentin-1 a promising candidate for the treatment of inflammation-induced osteoporosis.

Herein, we provided the first evidence that depletion of omentin-1 could induce a similar phenotype like inflammation-mediated osteoporosis in mice, as defined by abnormally elevated inflammatory cytokines, increased osteoclast formation and bone destruction, as well as impaired osteogenic activities. Using an inflammatory cell model induced by TNF-α, we determined that recombinant omentin-1 was capable of impairing the production of pro-inflammatory mediators in the TNF-α-activated macrophages, and suppressing their anti-osteoblastic and pro-osteoclastic effects. Moreover, we found that systemic administration of adenoviral-delivered omentin-1 could potently inhibit inflammation-induced osteoporotic bone loss in vivo. Thus, besides acting as a bone resorption inhibitor via reducing RANKL expression in osteoblasts,^9,10^ omentin-1 is also able to exert bone protective effects by inhibiting the inflammatory responses of macrophages, indicating a dual functional role of omentin-1 in bone homeostasis. We also tested whether depletion of omentin-1 affects the functions of the non-skeletal systems, such as liver, kidney, etc. The results showed no significant differences in the corresponding indicators between *omentin-1*^−/−^ mice and their wild-type littermates, suggesting that the function of these organs may not be affected by omentin-1 depletion, which warrants further investigation.

In this study, the inflammatory process is induced by injecting magnesium silicate subcutaneously into the back of the mice at distant sites from the skeleton, to induce acute phase response following chronic inflammation and granuloma formation. In agreement with previous data,^30,31^ we found that the magnesium silicate-treated mice exhibited a significant increase in the expression of various pro-inflammatory factors (including IL-1β, IL-6, and TNF-α) and an accumulation of pro-inflammatory macrophages in the bone marrow, as compared with the control group. The changes in pro-inflammatory mediator levels in blood were not as obvious as in bone, as only the serum concentration of IL-1α was found to be markedly enhanced after magnesium silicate treatment, suggesting that the inflammatory responses in bone marrow is stronger than that in other tissues, maybe because more inflammatory cells and their progenitors living in the bone marrow. As expected, magnesium silicate treatment resulted in a prominent reduction of bone mass and cortical bone formation, as well as deterioration of trabecular bone microstructure in mice. The outcome might be attributable to a combination of depressed osteoblast-mediated bone formation and increased osteoclast-mediated bone resorption, as evidenced by OCN and TRAP staining. This is also the case in humans with chronic inflammatory diseases.^32^

Although the pathogenesis of osteoporosis is multi-factorial, the local and systemic excessive production of pro-inflammatory cytokines is believed to play critical roles in the occurrence and development of osteoporosis. These cytokines, such as IL-1α, IL-1β, IL-6, and TNF-α, disturb the homeostasis between bone-forming osteoblasts and bone-resorbing osteoclasts. Studies have revealed that these cytokines are capable of effectively stimulating RANKL production in osteoblasts and stromal cells, which enhance osteoclasts formation for degradation of the adjacent bone.^33,34^ Evidence has also shown that IL-6 and TNF-α can suppress osteoblast differentiation and activity via downregulating BMP and TGF-β signaling pathways.^35^ In our study, we found that omentin-1 depletion in mice induced a local and systemic increase in the production of these pro-inflammatory cytokines, and overexpression of omentin-1 was effective to inhibit the magnesium silicate-induced upregulation of IL-1α, IL-1β, IL-6, and TNF-α in the bone tissue and/or in the blood stream of mice. In addition, we found that omentin-1 overexpression could markedly reduce the magnesium silicate-induced increase of pro-inflammatory macrophages (M1 phenotype) and enhance the amount of anti-inflammatory macrophages (M2 phenotype). These data, along with a recent study showing that omentin-1 is able to promote anti-inflammatory M2 phenotype during differentiation of monocytes into macrophages,^36^ suggest that omentin-1 may shift the macrophage phenotype overwhelmingly to M2 rather than M1, thus reducing the production of pro-inflammatory factors and maintaining normal bone mass. Since bacterial translocation in the gut and defense mechanism against intestinal bacterial permeation play crucial roles in chronic inflammation,^37^ and the protein sequence of omentin-1 is determined to be identical to intelectin-1, a galactofuranose-binding protein that acts as a host defense against invading pathogenic microorganisms,^38^ the inhibition of bacterial translocation may also be an important mechanism by which omentin-1 prevents inflammation and protects bone. It should to be noted that the omentin-1 knockout mice also had a much higher level of IL-10, an anti-inflammatory cytokine able to inhibit bone resorption.^13,14^ This may be a compensation reaction to inhibit the excessive inflammation caused by omentin-1 deficiency.

In summary, our findings demonstrate that omentin-1 plays an essential role in the maintenance of normal bone mass and is able to alleviate magnesium silicate-induced inflammation and osteoporotic bone loss. The underlying mechanism may be the downregulation of the production of pro-inflammatory mediators in macrophages, as omentin-1 can inhibit the inflammatory responses of the activated macrophages and impair their anti-osteoblastic and pro-osteoclastic effects. Our study suggests that omentin-1 can be used as a therapeutic agent for the prevention and treatment of inflammatory bone diseases.

## Materials and methods

### Animals and treatments

Animal care and experimental procedures were approved by the Laboratory Animal Management Committee of Central South University and performed in accordance with the guidelines of the National Institutes of Health Guidelines for the Care and Use of Laboratory Animals.

The global *omentin-1* gene knockout (*omentin-1*^−/−^) C57BL/6 mice were generated by Cyagen Biosciences Inc. (Guangzhou, China) using TALEN technology. TALEN target sites were designed in exon 4 of the mouse *omentin-1* gene and one base (A) was inserted into the sequence of *omentin-1* gene in one strand, causing a frameshift mutation and an early stop of omentin-1 protein translation. The mRNA transcribed from targeted allele with frameshift undergoes nonsense-mediated decay. The target TALEN sequences of the *omentin-1* gene were: left, 5′-TTTCCTGCGCACGAAGAA-3′, and right, 5′-TCATGTCACAGAAGGTCT-3′. The knockout mice were produced by microinjecting TALEN mRNAs into fertilized eggs obtained by superovulation of female C57BL/6 mice mating with the males of the same strain. Genomic DNA was extracted from tail tips of founder mice (F0) and genotyped by PCR followed by DNA sequencing analysis. PCR was carried out using the following primers: forward, 5′-CTGCACAGAGGAAGACTGTGGACC-3′, and reverse, 5′-AAAGGTGTTGTAGTTGGCCCAGTTG-3′. The positive founders were breeding to the next generation which was genotyped by PCR and DNA sequencing analysis. The small intestines, the organ showing the highest expression of omentin-1 in mice,^11,12^ were collected from *omentin-1*^−/−^ mice and their wild-type littermates for western blotting to verify the depletion of omentin-1. Mice were fed on a commercially available basal diet (SCXK2014-0002; SLAC Laboratory Animal Ltd. Co., Shanghai, China). Body weights were measured monthly from birth to 5 months of age. Three-month-old male omentin-1^−/−^ mice and their wild-type littermates were used for further analyses. Food and water intake were recorded daily. Blood glucose was measured from mouse tail vein using glucometer. Blood samples were collected by cardiac puncture immediately after euthanasia and serum samples were obtained by centrifugation (1 000×*g*, 15 min). The levels of serum IL-1α, IL-1β, IL-6, TNF-α, OCN, CTX-I, omentin-1, insulin, ALT, and AST were tested using commercially available ELISA kits from Multi Sciences LTD (Hangzhou, China) or Elabscience (Wuhan, China). The levels of serum iron, creatinine and blood urea nitrogen were measured using commercial kits from Jiancheng (Nanjing, China) and Solarbio (Beijing, China), respectively. Bone samples were also removed for further analysis.

Three-month-old male C57BL/6 wild-type mice (weighing 25–30 g) were used for investigating the therapeutic effect of omentin-1 on inflammation-induced bone loss. Experimental inflammatory osteoporosis models were induced by a single-subcutaneous injection of magnesium silicate (0.2 g, dissolved in 2 mL of sterile saline) into the back of mice with 8 injection sites, and controls received an equal volume of saline. Subsequently, the adenovirus encoding omentin-1 (Ad-Omt) or control adenovirus encoding β-galactosidase (Ad-Gal) at the dose of 1 × 10^8^ plaque-forming units were intravenously injected into the tail vein of mice and the injection was administered one time per week. Three weeks later, bone and blood samples from mice were collected and processed for further experiments.

### µCT analysis

The right femora dissected from mice were fixed overnight in 4% paraformaldehyde and analyzed with high-resolution µCT (Skyscan 1176) as described previously.^39,40^ The scanner was set at a voltage of 50 kV, a current of 400 µA and a resolution of 8.88 µm per pixel, respectively. The image reconstruction software (NRecon), data analysis software (CTAn v1.11), and three-dimensional model visualization software (µCTVol v2.2) were used to analyze the parameters of the distal femoral metaphyseal trabecular bone. Trabecular bone region of interest (ROI) was drawn starting from 0.15 mm proximal to distal epiphyseal growth plate and extended proximally for 0.4 mm length. The trabecular bone volume per tissue volume (BV/TV), trabecular thickness (Tb. Th), trabecular number (Tb. N) and trabecular separation (Tb. Sp) were measured. Cross-sectional images of femur were used to perform three-dimensional histomorphometric analysis of cortical bone. For cortical bone, the ROI selected for analysis was of 10% of femoral length in mid-diaphysis of the femur to assess periosteal perimeter (Ps. Pm) and cortical thickness (Ct. Th).

### Immunohistochemical analyses

Fresh femora were dissected from mice, fixed in 4% paraformaldehyde for 48 h, decalcified in 10% EDTA for 21 days, and then embedded in paraffin. Samples were cut into 5-µm-thick longitudinally oriented sections and stained for OCN and TRAP in order to detect osteogenic and osteoclastic activities. The extent of inflammatory responses in the bone tissues was assessed by immunohistochemical analysis of IL-1β, IL-6, and TNF-α. CD86 and CD206 staining was performed to detect the presence of macrophages in bone tissues from mice treated with or without magnesium silicate in the presence and absence of Ad-Omt treatment. Positively stained cells or relative staining intensity were measured in three random visual fields of distal metaphysis per femur in three sequential sections per mouse in each group as previously described.^40^ Images were acquired with an optical microscope (CX31; Olympus, Hamburg, Germany). TRAP staining kit was purchased from Sigma-Aldrich (St. Louis, MO, USA). Anti-IL-1β, anti-IL-6, and anti-TNF-α were obtained from ProteinTech (Chicago, USA). Anti-CD86 was obtained from Novus Biologics (Littleton, USA). Anti-CD206 and all the secondary antibodies were purchased from Abcam (Cambridge, Britain).

### CFU-F and CFU-GM assays

Bone marrow cells were obtained from the femurs and tibiae of mice by flushing with a 22-gauge needle and then were resuspended. After rinsing by centrifugation, cells were resuspended and counted. A total of 5 × 10^5^ marrow cells were plated into six well plates in 2 mL of culture medium for both the CFU-F and CFU-GM assays. Triplicate cultures were established. After 3 days of adhesion, unattached cells were removed and fresh medium was added to the adherent cells. The whole medium was changed every 3 days. On day 14, colonies were stained with 0.1% crystal violet (Solarbio). Colonies consisting of >50 cells were defined as CFU-F or CFU-GM. The colony- forming efficiency was determined by counting the number of colonies per 5 × 10^5^ marrow cells plated. CFU-Fs were cultured in α-MEM medium (Hyclone; Thermo Scientific, USA) with 2 mmol·L^-1^ glutamine (Gibco BRL, Grand Island, USA), 100 U·mL^-1^ penicillin (Gibco), 100 U·mL^-1^ streptomycin (Gibco) and 20% fetal bovine serum (FBS; Gibco). CFU-GMs were cultured in α-MEM medium with 20 ng·mL^-1^ M-CSF (Novus Biological Inc., Littleton, CO, USA), 2 ng·mL^-1^ GM-CSF (Novus), 2 mmol·L^-1^ glutamine, 100 U·mL^-1^ penicillin, 100 U·mL^-1^ streptomycin, and 20% FBS.

### Culture of MSCs and macrophages

MSCs were prepared from male mouse strain C57BL/6-Tg(UBC-GFP)30Scha/J (Jackson Laboratory). The macrophage cell line RAW264.7 was purchased from American Type Culture Collection (Rockville, MD, USA). The cells were cultured in high-glucose DMEM (Gibco) containing 10% FBS, 100 U·mL^-1^ penicillin and 100 U·mL^-1^ streptomycin at 37 °C in 5% CO_2_ in a humidified environment. Non-adherent cells were removed, and the adherent cells were passaged after becoming 80% confluent.

### Preparation of CM from inflammatory macrophages

An inflammatory cell model was established by treating RAW264.7 macrophages with recombinant TNF-α (10 ng·mL^-1^; Pepro Tech, Rocky Hill, USA) for 30 min. Subsequently, the culture medium was replaced to remove residual TNF-α and the cells were then incubated in fresh medium supplemented with recombinant omentin-1 (300 μg·mL^-1^; RayBiotech, Guangzhou, China) or an equal volume of diluents (PBS) for 24 h. The CM from inactivated (treated with PBS), TNF-α-activated, or TNF-α + omentin-1-treated RAW264.7 macrophages were obtained and centrifuged at 2 000×*g* for 10 min to remove dead cells and cellular debris. The supernatant was collected and stored at −80 °C or used for downstream experiments.

### ELISA

Serum from all the animal groups and the CM from RAW264.7 macrophages were collected and stored at −80 °C until they were thawed for the assay. The concentrations of IL-1α, IL-1β, IL-6, and TNF-α in serum or CM were measured using ELISA kits from Multi Sciences LTD. (Hangzhou, China). The levels of serum OCN, CTX-I, omentin-1, insulin, ALT, and AST were assessed using ELISA kits from Elabscience (Wuhan, China). All procedures were performed according to the manufacturer’s instructions.

### Osteogenesis induction

MSCs (1.0 × 10^4^ per well) were plated in 48-well plates and cultured in high-glucose *DMEM* supplemented with 10% FBS for 24 h. Next, the cells were rinsed with PBS and the medium was replaced with osteogenic differentiation medium (Cyagen Biosciences Inc, Santa Clara, USA) supplemented with CM obtained from RAW264.7 cells stimulated by TNF-α, TNF-α + omentin-1, or an equal volume of PBS. MSCs cultured in DMEM + 10% FBS were served as the negative control. Half of the medium was changed every 3 days. ALP staining, ARS staining, and gene expression analysis were performed at the indicated times.

### ALP and ARS staining

After 3 days of osteogenic induction, the differentiated MSCs were washed with PBS, fixed with 4% paraformaldehyde for 2 min, and stained for ALP activity with a commercially available ALP staining kit (Beyotime, Shanghai, China). After 14 days of differentiation, the cells were washed with PBS, fixed with 4% paraformaldehyde for 10 min, and then incubated with 2% ARS solution (Solarbio) according to the instructions provided by the manufacturer. After washing with PBS, the stained cells were examined using an inverted microscope (Leica DMI6000B, Solms, Germany).

### TRAP staining

RAW264.7 cells were plated in 48-well plates at a concentration of 1.5 × 10^4^ per well and incubated with 100 ng·mL^-1^ RANKL and CM obtained from RAW264.7 macrophages stimulated by TNF-α, TNF-α + omentin-1, or an equal volume of PBS. The negative control culture was grown in high-glucose DMEM supplemented with 10% FBS. Half of the medium was changed every 3 days. After 6 days of induction, the cells were washed with PBS and fixed with 4% paraformaldehyde for 10 min. After washing with distilled water, the cells were stained with TRAP using a commercially available kit (Sigma). TRAP^+^ multinucleated cells (MNCs) showing more than three nuclei were considered to be osteoclasts. The number of osteoclasts was counted under an inverted microscopy (Leica).

### qRT-PCR analysis

Total RNA was extracted using TRIzol Reagent (Invitrogen, Carlsbad, CA) and cDNA was synthesized from 1 μg of total RNA by using the Revert Aid First Strand cDNA synthesis kit (Fermentas Life Sciences, Burlington, Canada). Then, the cDNA was amplified with FastStart Universal SYBR Premix ExTaq^TM^ II (Takara Biotechnology, Japan) in an ABI PRISM® 7900HT System (Applied Biosystems, Foster City, USA). The relative standard curve method (2^–△△CT^) was used to determine the relative gene expression and GAPDH was used as a housekeeping gene for internal normalization. The PCR primers used in this study were as follows: *IL-1α*: forward, 5′-CGAAGACTACAGTTCTGCCATT-3′, and reverse, 5′-GACGTTTCAGAGGTTCTCAGAG-3′; *IL-1β:* forward, 5′-GAAATGCCACCTTTTGACAGTG-3′, and reverse, 5′-TGGATGCTCTCATCAGGACAG-3′; *IL-6*: forward, 5′-TAGTCCTTCCTACCCCAATTTCC-3′, and reverse, 5′-TTGGTCCTTAGCCACTCCTTC-3′; *Runx2*: forward, 5′-GACTGTGGTTACCGTCATGGC-3′, and reverse, 5′-ACTTGGTTTTTCATAACAGCGGA-3′; *OCN*: forward, 5′-CTGACCTCACAGATCCCAAGC-3′, and reverse, 5′-TGGTCTGATAGCTCGTCACAAG-3′; *ALP*: forward, 5′-CCAACTCTTTTGTGCCAGAGA-3′, and reverse, 5′-GGCTACATTGGTGTTGAGCTTTT-3′; *Trap*: forward, 5′-TGGTCCAGGAGCTTAACTGC-3′, and reverse, 5′-GTCAGGAGTGGGAGCCATATG-3′; *RANKL*: forward, 5′-GCCATTTGCACACCTCACCA-3′, and reverse, 5′-GCCGAAAGCAAATGTTGGCG-3′; Mmp9: forward, 5′-ACCCGAAGCGGACATT-3′, and reverse, 5′-GGCATCTCCCTGAACG-3′; Ctsk: forward, 5′-GCGGCATTACCAACAT-3′, and reverse, 5′-CTGGAAGCACCAACGA-3′; ATP6V0d2: forward, 5′-AGCAAAGAAGACAGGGAG-3′, and reverse, 5′-CAGCGTCAAACAAAGG-3′; Oscar: forward, 5′-GGTCCTCATCTGCTTG-3′, and reverse, 5′-TATCTGGTGGAGTCTGG-3′; NFATc1: forward, 5′-CAGTGTGACCGAAGATACCTGG-3′, and reverse, 5′-TCGAGACTTGATAGGGACCCC-3′; GAPDH: forward, 5′-CACCATGGAGAAGGCCGGGG-3′, and reverse, 5′-GACGGACACATTGGGGGTAG-3′.

### Western blotting

Protein extracts were separated by SDS-PAGE and blotted onto polyvinylidene fluoride membranes (Immobilon P, Millipore, Billerica, USA). Blots were blocked with 5% non-fat milk in TBST for 1 h at room temperature. The membranes were then incubated with primary antibodies at 4 °C overnight and with the horseradish peroxidase-conjugated secondary antibodies at 37 °C for 1 h. The anti-omentin-1 antibody was purchased from R&D system (Minneapolis, MN, USA). Anti-IL-1β and anti-IL-6 were obtained from ProteinTech (Chicago, USA). Anti-β-actin and all the secondary antibodies were purchased from Cell Signaling Technology (Danvers, MA, USA). Protein bands were visualized using enhanced chemiluminescence reagent (Thermo Fisher Scientific, Waltham, USA) and imaged by ChemiDoc XRS Plus luminescent image analyser (Bio-Rad, Hercules, CA, USA).

### Statistical analysis

All data are presented as means ± standard deviation (s.d.). Two tailed Student’s *t*-test was used to compare means between two groups. All statistical analysis was conducted using GraphPad Prism software and *P* < 0.05 were considered statistically significant.

## Electronic supplementary material

Supplementary Figure 1-5(DOCX 1091 kb)
