# Supplementary material for: Omentin-1 prevents inflammation-induced osteoporosis by downregulating the pro-inflammatory cytokines
Source: Bone Res. 2018 Mar 30;6:9. doi: 10.1038/s41413-018-0012-0 (PMC5876344; doi:10.1038/s41413-018-0012-0)
Supplement: Supplementary file 1 — Supplementary Figure 1-5(DOCX 1091 kb) [file 41413_2018_12_MOESM1_ESM.docx]

**Supplementary Material**

**Omentin-1** **prevents inflammation-induced osteoporosis** **by down-regulating the pro-inflammatory** **cytokines**

Shan-Shan Rao^1,6*^, Yin Hu^1,4*^, Ping-Li Xie^7^, Jia Cao^1^, Zhen-Xing Wang^1^, Jiang-Hua Liu^1,4^, Hao Yin^1,4^, Jie Huang^1,4^, Yi-Juan Tan^1^, Juan Luo^1^, Ming-Jie Luo^8^, Si-Yuan Tang^8^, Tuan-Hui Chen^1^, Ling-Qing Yuan^9^, Er-Yuan Liao^9^, Ran Xu^9^, Zheng-Zhao Liu^1^, Chun-Yuan Chen^1#^ and Hui Xie^1-5,10#^


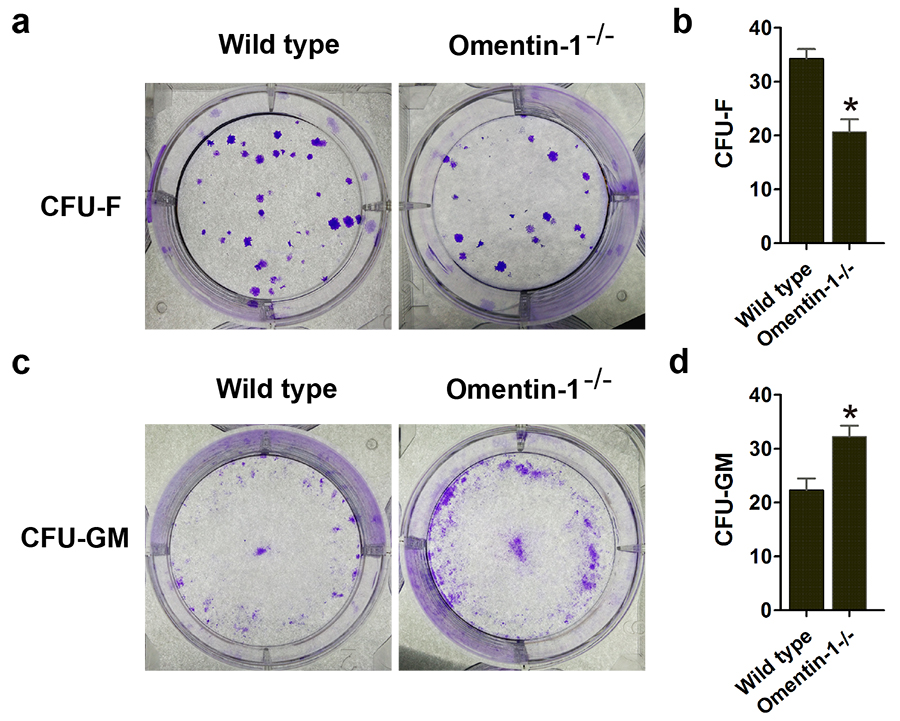


**Supplementary Figure 1.** **Omentin-1 depletion** **reduces CFU-F colonies and increases CFU-GM colonies.** (**a**) Representative images of crystal violet-stained CFU-F colonies in six-well plates. (**b**) Quantitative analysis of the numbers of CFU-F colonies per 5 × 10^5^ marrow cells plated. n = 3 per group. (**c**) Representative images of crystal violet-stained CFU-GM colonies in six-well plates. (**d**) Quantitative analysis of the numbers of CFU-GM colonies per 5 × 10^5^ marrow cells plated. n = 3 or 4 per group. Data are shown as the mean ± s.d. ******P* < 0.05 compared with the control (wild type) mice**.**


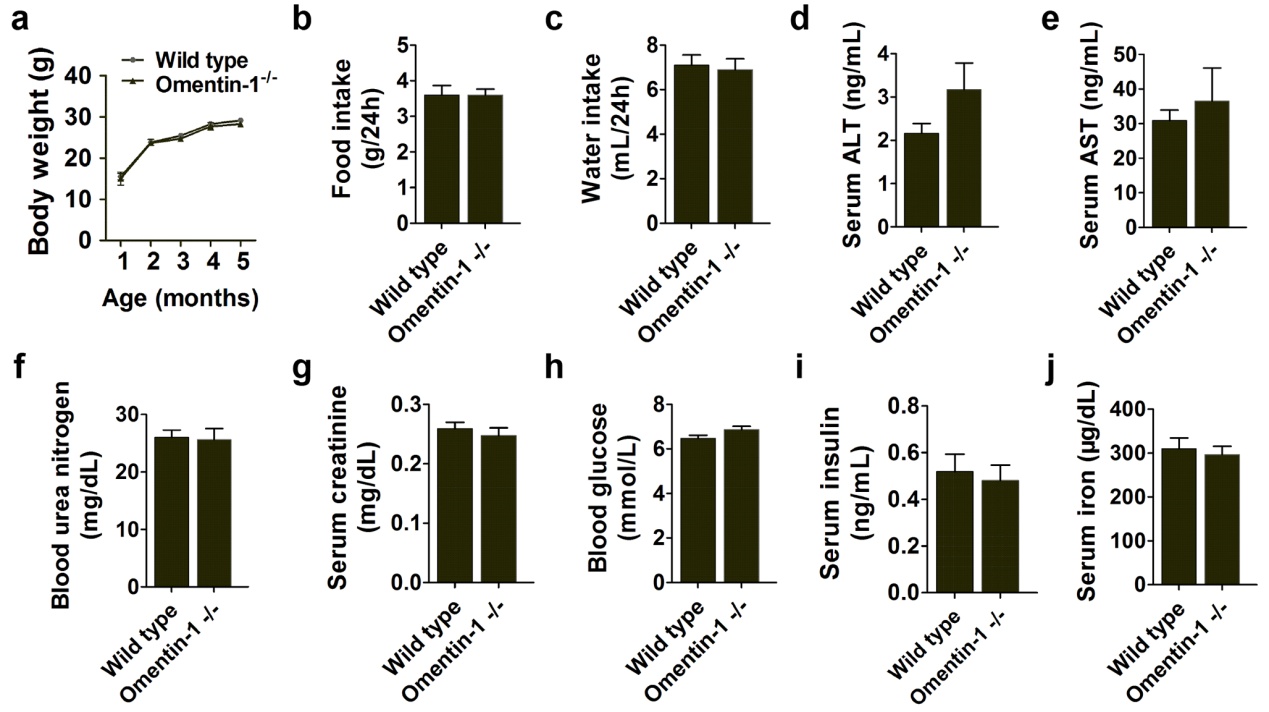


**Supplementary Figure 2. Omentin-1** **depletion** **induces non-significant effects on non-skeletal systems.** (**a**) Body weights of *omentin-1*^–/–^ mice and their wild-type littermates at indicated times. n = 6 per group. (**b-c**) Daily food and water intake of *omentin-1*^–/–^ and wild type mice. n = 10 per group. (**d-j**) The concentrations of serum ALT, AST, blood urea nitrogen, creatinine, glucose, insulin and iron in *omentin-1*^–/–^and wild type mice. n = 8-16 per group. Data are shown as the mean ± s.d. ******P* < 0.05 compared with the wild type mice**.**


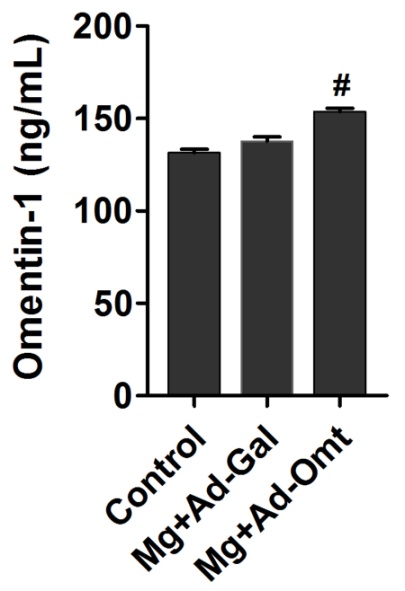


**Supplementary Figure 3. The efficiency of Ad-Omt is confirmed by ELISA assay for serum omentin-1.** n = 7-8 per group. Data are shown as the mean ± s.d. ******P* < 0.05 compared with the control group, **^#^** *P* < 0.05 compared with the magnesium silicate + Ad-Gal (Mg + Ad-Gal) group.


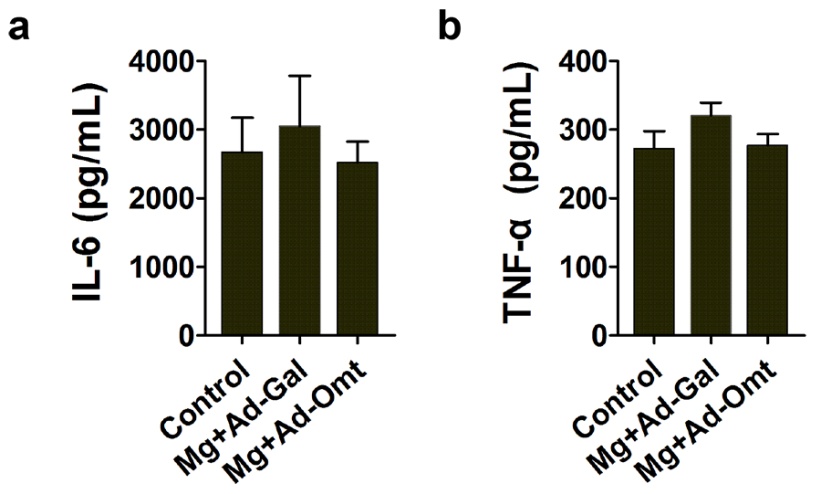


**Supplementary Figure 4. Magnesium silicate and omentin-1** **induce non-significant effects in the serum levels of IL-6 and TNF-α.** (**a-b**) The serum concentration of IL-6 (**a**) and TNF-α (**b**) in mice receiving different treatments, as analyzed by ELISA. n = 7-9 per group. Data are shown as the mean ± s.d. ******P* < 0.05 compared with the control group, **^#^** *P* < 0.05 compared with the Mg + Ad-Gal group.


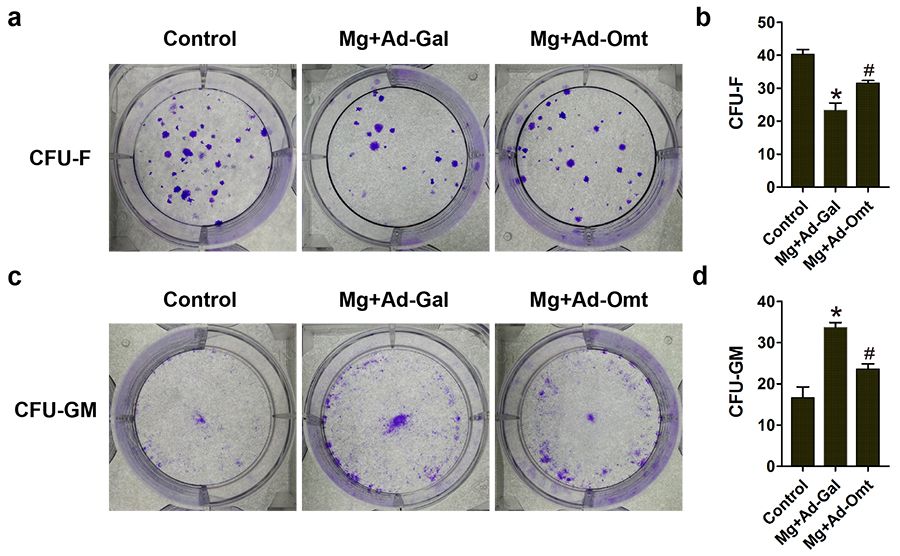


**Supplementary Figure 5. Omentin-1 reverses the magnesium silicate-induced reduction of CFU-F colonies and the stimulation of CFU-GM colonies.** (**a**) Representative images of crystal violet-stained CFU-F colonies in six-well plates. (**b**) Quantitative analysis of the numbers of CFU-F colonies per 5 × 10^5^ marrow cells plated. n = 3 per group. (**c**) Representative images of crystal violet-stained CFU-GM colonies in six-well plates. (**d**) Quantitative analysis of the numbers of CFU-GM colonies per 5 × 10^5^ marrow cells plated. n = 3 per group. Data are shown as the mean ± s.d. ******P* < 0.05 compared with the control group, **^#^***P* < 0.05 compared with the Mg + Ad-Gal group.
